# Challenges for Drug Repurposing in the COVID-19 Pandemic Era

**DOI:** 10.3389/fphar.2020.588654

**Published:** 2020-11-06

**Authors:** Janet Sultana, Salvatore Crisafulli, Flic Gabbay, Elizabeth Lynn, Saad Shakir, Gianluca Trifirò

**Affiliations:** ^1^ Department of Biomedical and Dental Sciences and Morphofunctional Imaging, University of Messina, Messina, Italy; ^2^ TranScrip, Reading, United Kingdom; ^3^ Drug Safety Research Unit, Southampton, United Kingdom; ^4^ School of Pharmacy and Biomedical Sciences, University of Portsmouth, Portsmouth, United Kingdom

**Keywords:** drug repurposing, drug repositioning, COVID-19, pandemic, coronavirus

## Abstract

The coronavirus disease (COVID-19) pandemic has affected an estimated 16 million persons and caused 0.6 million deaths worldwide by September 2020. The pandemic has led to a rush to repurpose existing drugs, although the underlying evidence base is of variable quality. The improving knowledge of the virology and clinical presentation of COVID-19 is leading to a broadening pool of potential pharmacological targets. The aim of this review is to describe regulatory and pharmacological aspects of drug repurposing and to identify drugs proposed for repurposing in COVID-19 based on registered clinical trials, discussing the evidence to support their use in the treatment of this disease. The challenges of the correct interpretation of existing pre-clinical/clinical evidence as well as the generation of new evidence concerning drug repurposing in COVID-19 will also be discussed.

**Clinical Trial Registration:** https://clinicaltrials.gov, identifier NCT04321174, NCT04342663, NCT04280705, NCT04244591, NCT04359329, NCT04348695, NCT04304313, NCT043505931

## Introduction

To date, the global coronavirus disease 2019 (COVID-19) pandemic, caused by the severe acute respiratory syndrome coronavirus 2 (SARS-CoV-2), has led to more than 16 million infected patients and more than 600,000 deaths worldwide by September 2020 (European Centre for Disease Prevention and Control, 2020). To date, only remdesivir, an investigational antiviral compound, has received a conditional marketing authorization from the European Commission for the treatment of COVID-19 in adults and adolescents from 12 years of age with pneumonia who require supplemental oxygen (European Medicines Agency, 2020a). Remdesivir has also been approved in Japan (Gilead, 2020) and has received Emergency Use Authorization in the United States from the Food and Drug Administration (Food and Drug Administration, 2020a). No other treatments have been approved to date.

There is great interest in drug repurposing (also known as repositioning or rediscovery) to accelerate the identification of drugs that can cure or prevent COVID-19. The value of drug repurposing is to speed up the traditional process of drug discovery by identifying a novel clinical use for drugs that have already proven to be safe and effective in humans and are approved for other indications. This strategy can also reduce the costs required for the development of new drugs, with notable savings in preclinical phase I and II (Pushpakom et al., 2018). The rationale of drug repurposing lies in the fact that the same molecular pathways may be involved in different diseases (Oprea et al., 2011). One of the key drivers for the repositioning of drugs is the serendipitous discovery of pharmacological activity on new targets, which would then suggest that a new possible indication of use (Pushpakom et al., 2018). Some classical examples of serendipity-based drug repurposing are thalidomide, originally developed for the treatment of morning sickness in pregnant women and now used in multiple myeloma (Jacobson, 2000), sildenafil, initially conceived for the treatment of angina and hypertension and today used for the treatment of erectile dysfunction (Ghofrani et al., 2006) and amantadine, an antiviral which was originally indicated for influenza and was then used to treat Parkinson’s disease (Lee and Kim, 2016).

Repurposing has several implications in the drug regulatory setting as well as in the scientific setting, especially if it occurs during a public health emergency such as the COVID-19 pandemic. In this review, challenges for drug repurposing in the COVID-19 pandemic era will be described. In addition, drugs proposed as candidates for repurposing in COVID-19 will be identified and the rationale behind their use will be discussed.

## Drug Repurposing From a Regulatory Perspective

Repurposing officially falls into a number of categories, bearing in mind that physicians in most countries have the right to use drugs outside the existing approved label and frequently do. However, “off-label” use is often frowned upon by regulatory drug agencies and scientific societies, as the effectiveness and safety of drugs for off-label indications may not be established. In most cases, to have a new indication approved, substantial investment is required. A patent is essential for the sponsor of the development program to ensure return on investment with sales. Patents based on “product” or “composition-of-matter” not only give the patentee the right to exclude others from making and selling the drug for the same purpose as the patentee but also block the marketing of any new use that another party discovers. The patent process also runs alongside extensions of exclusivity built into some regulatory processes. In contrast, so-called “use” patents protect a selected therapeutic use. Repurposed drugs fall into four categories based on those described by the Discovering New Therapeutic Uses for Existing Molecules initiative of National Institute of Health (NIH) through the National Center for Advancing Translational Sciences (NCATS) (https://ncats.nih.gov/ntu): 1) Therapeutic assets (of any modality) with remaining patent life but never approved for human use; 2) Therapeutics with remaining patent life that are currently approved for one or more indication(s) but have potential use in others; 3) Therapeutic assets with no patent life that are not currently marketed because they were either never approved or were withdrawn; 4) Therapeutics with no patent life currently manufactured by generic companies, approved for certain indications, and available by prescription from healthcare providers. The first two can be developed by the patent holder or licensed to another company for development; the second two can be developed by establishing a “use” if it fits patentable criteria of being sufficiently novel, useful, and non-obvious. All types of products must meet all the normal benefit-risk assessment requirements of drug regulation based on the evidence of quality, safety and efficacy and with appropriate prescriber information, if they are to be distributed to the public. In addition, compliance with health technology assessment (HTA) to oversee pricing and reimbursement and the way the product is positioned in national guidance for standard of care is becoming increasingly important in all major countries and, to a greater or lesser extent, government controlled.

Regulation and HTA require one or more clinical trials for the new indication unless it is an exceptional situation. One such exceptional situation concerns extremely rare conditions, such as anthrax and Ebola, which can be registered on animal data and human safety data from healthy volunteers or other indications. An example of this is Anthrasil^™^, Anthrax Immune Globulin Intravenous (Human), approved by FDA in 2015 to treat patients with inhalational anthrax in combination with appropriate antibacterial drugs already registered under the animal rule. Another exceptional situation concerns a product currently used for the indication. This applies in Europe as “well-established use”. When an active ingredient of a medicine has been used for more than 10 years its efficacy and safety may be considered well-established. In such cases, marketing authorization may be based on results from the scientific literature. There are also regulatory mechanisms for the active ingredient to be used at a different dose (hybrid applications) and an example of this is dexamethasone registered by EMA as Neofordex^™^ under well-established use in 2016 for multiple myeloma. Most products, however, will need more than a single clinical trial to establish the new indication for repurposing. If the indication is completely new, repurposed drugs normally start with preclinical pharmacology, including animal models and safety, aimed at defining dose and length of treatment, followed by a translational medicine and a clinical program, similarly to the approval pathway of a new drug.

Regulatory decisions are made on benefit-risk balance and in a rapidly evolving pandemic two issues come in to play, namely the speed of drug approval and the urgency of clinical needs. The speed of developing treatments to the point of approval is key to protect patients from the infection as quickly as possible. Drug development of new biologicals or chemicals, other than vaccines, takes a minimum of 2–3 years even if there is a candidate that has shown efficacy in animal trials and have scaled down clinical trials to 6–12 months. Pre-clinical activities of manufacturing to establish product quality and the necessary safety studies normally take at least 2 years before clinical trials can start. Repurposed drugs, however, could appear on the market after simply completing one or more clinical trials. As the medical need for a drug increases and more patients die from the disease, the benefit of the drug increases in the assessment of benefit-risk balance. For new biologicals or small-molecule drugs the risk element is not yet established in clinical medicine and it takes time to accumulate enough patients for this purpose under the new indication, even if efficacy can be established quickly. A potential way is to accelerate the generation of evidence to separate safety trials from efficacy trials, as in medicines and vaccines registered under the animal rule (Food and Drug Administration, 2015; European Medicines Agency, 2018). In United States there is specific guidance for developing products where efficacy can only be established by the animal rule (FDA, 2015) but in Europe the guidance is included within a specific guidance for types of development such as the vaccine guideline (European Medicines Agency, 2018). The alternative is to use repurposed drugs where the human risk profile is known.

In situations such as the COVID-19 pandemic, regulatory agency decisions may have to accelerate their decision process, thus collaboration and communication between national regulatory and HTA agencies becomes essential. The aim should be to reach peak acceleration of review without compromising the benefit-risk balance assessment. Some changes in processes may be continued after the pandemic and it could be argued that they have demonstrated general improvement in process efficiency such as rolling review in EMA which has been planned to be extended in their strategy up to 2025 (European Medicines Agency, 2020b). Some compromises in process, however, such as monitoring and audit of clinical trial sites is unique to lockdown and is not planned to continue.

All drug regulatory agencies have committed to speeding up regulations and all are collaborating through the International Coalition of Medicines Regulatory Authorities (ICMRA) whose website contains valuable information on COVID-19 trial programmes for medicines and vaccines in development. Each individual agency has also issued guidance which not only speeds the time to obtain clinical trial approval but also makes scientific advice more rapidly accessible, dropping normal agency timelines and conducting video meetings. With regard to minimal evidence, all major agencies (Europe, United Kingdom, Japan and the United States) already had robust processes in place for accelerated approvals which require minimal evidence. This is illustrated by remdesivir, a repurposed drug that was originally developed for Ebola. Remdesivir received Japanese authorization under the Exceptional Approval Pathway, Emergency Use Authorization in the USA and in the United Kingdom access was granted under the Early Access to Medicine Scheme. Conditional approval for remdesivir by EMA for use to treat COVID-19 in adults and adolescents with pneumonia requiring supplemental oxygen was given under the new accelerated pathway under guidance issued in May 2020 (EMA, 2020). The main efficacy study assessed was NIAID-ACTT-1 (Beigel et al., 2020) involving 1,063 hospitalized patients with COVID-19 (120 with mild to moderate disease and 943 with severe disease) showed that Veklury^™^ (remdesivir) can speed up the recovery time in some patients, allowing them to spend less time in hospital or on treatment. The further clinical data required by EMA is published in their web site (European Medicines Agency, 2020c; European Medicines Agency, 2020d). In both United States and EU pediatric data planning is also required. It is worth noting that although remdesivir use was supported by *in vivo* studies due to its initial indication for Ebola virus, it was trialed in a Phase III clinical trial for Ebola,with that safety data being also used for the evaluation of the Emergency Use Approval in the United States (Mulangu et al., 2019).

The expedited drug development and regulatory decision-making processes may come at the cost of complete drug safety and effectiveness data. Indeed, the drug development process, which usually takes 12–15 years, is reduced to 12–18 months or less. Normally, conducting Phase III clinical trials is a lengthy process; reducing the time needed so drastically can only be achieved by conducting shorter, fewer or in extreme cases, no Phase III clinical trials. This can occur in a pandemic when the recruitment stage coincides with a period of low incidence of the disease, such as the trial of remdesivir by Wang et al., which was stopped early because there were not enough infected patients available in China to reach trial recruitment targets (Wang et al., 2020). Therefore, during a pandemic, drugs can be licensed with less information available than would normally be acceptable. In the case of COVID-19 it depends on the prevalence of the disease but repurposed products could be licensed on the basis of their benefit risk evaluations for other indications, biomarker data and limited phase III studies for the COVID-19 indication. Emergency Use Authorization (FDA), Conditional Marketing Authorization (EMA) or approval under the Early Access to Medicines Scheme (MHRA) for COVID-19 were based on very limited clinical data. Even the full Marketing Authorization are likely to be granted on the basis of limited clinical data compared to the conventional licensing requirements. Therefore, real-world studies to monitor the safety and effectiveness post-licensing are necessary during a pandemic. Regulatory decisions are likely to be based on limited Phase III data or in some circumstances only on the results of Phase II trials with a conditional license to conduct studies in the post-marketing phase. It is well known that there are gaps in available safety information at the time of licensing for all medicinal products. This is the nature of clinical development which focuses on efficacy, and is the reason for post-authorization risk management plans which aim to address any safety uncertainties during the postmarking phase. Such uncertainties are significantly higher for products that have gone through expedited development and licensing. Hence, robust post-authorization studies with early and regular interim reporting are not only highly necessary but, in some cases, may be a condition of the Marketing Authorization.

## Drugs Candidates for Repurposing in COVID-19 Infection

To identify drugs being seriously considered for repurposing, clinicaltrials.gov, a repository for clinical trials, was searched for all registered clinical trials concerning COVID-19 on July 2, 2020. Studies aiming to evaluate drug safety were excluded, as were all studies concerning vaccines. Studies were included irrespectively of whether they were planned, ongoing or completed, and also irrespectively of whether the drugs were intended to prevent or treat COVID-19. For each drug being considered as a treatment in a clinical trial, the mechanism of action was identified through a literature search.

Overall, drugs currently being tested for repositioning in COVID-19 can be distinguished as 1) drugs potentially able to inhibit one or more steps of the coronavirus lifecycle (Table 1) and 2) drugs potentially able to counteract the effects of SARS-CoV-2 infection, such as the amplified immune response and the massive cytokine release, which both lead to severe complications such as coagulopathy and acute respiratory distress syndrome (ARDS) (Table 2). To date, only remdesivir has been approved to treat COVID-19.

**TABLE 1:** Overview of drugs proposed as potential inhibitors of one or more steps of SARS-CoV-2 lifecycle and undergoing experimental studies at 2nd July 2020– source: clinicaltrials.gov.

Pharmacological class	Drug	Proposed mechanism in the treatment of SARS-CoV-2 infection
Kinase inhibitors	Baricitinib	It could exert anti-viral effects by its affinity for AP2-associated protein AAK1, reducing SARS-CoV-2 endocytosis
	Imatinib	It accumulates in lysosomes resulting in some antiviral activities by lysosomal alkalization required for virus/cell fusion
Antibacterials	Doxycycline	It could reduce pro-inflammatory cytokines levels and chelate matrix metalloproteinases used for cell fusion and viral replication
Antidiabetic drugs	Dapagliflozin	During virus infection, serum lactate dehydrogenase level excessively rises. Dapagliflozin has been reported to reduce lactate levels by various mechanisms. It also reduces oxygen consumption in tissues and causes the use of glucose in the erobic pathway
	Linagliptin, sitagliptin	Since SARS-CoV-2 could use DPP4 receptor to invade cells, the inhibition of DPP4 could be useful in mild COVID-19 patients
Antimalarials	Artemisinin/artesunate	Anti-inflammatory activity, NF-κB-coronavirus effect and chloroquine-like endocytosis inhibition mechanism
	Atovaquone	It could inhibit SARS-CoV-2 through targeting of the viral RdRp or 3C-like protease
	Chloroquine, hydroxychloroquine	They showed interference with the glycosylation of ACE-2 receptors; they increase the pH of acidic cellular organelles, counteracting virus replication
	Mefloquine	It inhibited SARS-CoV-2 replication *in vitro* experimental models
Antitumorals	Plitidepsin	It could inhibit the multiplication and propagation of SARS-CoV-2
	Selinexor	It could inhibit the replication of SARS-CoV-2 and mediate anti-inflammatory and anti-viral effects
Antivirals	Atazanavir, danoprevir, darunavir	Potential SARS-CoV-2 protease inhibition
	Clevudine	It acts as a potent inhibitor of RdRp protein, preventing RNA replication
	Daclatasvir	It could target different proteins of the SARS-CoV-2 life cycle, affecting both viral RNA replication and virion assembly
	Emtricitabine	RNA synthesis nucleos(t)ide analogue inhibitors could have an effect against SARS-CoV-2 infection
	Favipiravir, galidesivir	They inhibit RdRp of RNA viruses, blocking SARS-CoV-2 replication
	Lopinavir/ritonavir	They could inhibit SARS-CoV-2 replication by blocking 3CL^pro^ and PL2^pro^ proteases
	Nelfinavir	It may bind to the S trimer structure inhibiting the membrane fusion process
	Nitazoxanide	It exerts antiviral effects through the phosphorylation of protein kinase activated by double-stranded RNA, which leads to an increase in phosphorylated factor 2-alpha, an intracellular protein with antiviral effects
	Oseltamivir	It could inhibit virus replication and virion release
	Remdesivir	It could inhibit the RNA synthesis of SARS-CoV-2
	Ribavirin	It could inhibit SARS-CoV-2 replication
	Sofosbuvir	It is a chain terminator for SARS-CoV-2 RNA polymerase. In human brain organoids, it protected from SARS-CoV-2-induced cell death
	Tenofovir alafenamide	It could inhibit SARS-CoV-2 RdRp
	Umifenovir	It could block trimerization of the spike glycoprotein, essential for host cell adhesion
Immunosuppressants	Cyclosporine	It can block viral replication and thus transcription of pro-inflammatory cytokines
	Leflunomide	In *vitro* studies have shown antiviral effects of leflunomide against SARS-CoV-2
	Sirolimus	It could block viral protein expression and virion release
	Tacrolimus	It inhibited SARS-CoV-2 replication *in vitro*
Interferons	Alpha and beta interferons	Interferons exhibit both direct inhibitory effects on viral replication and supporting an immune response to clear virus infection
	Peginterferon lambda-1A	It inhibits viral replication without and does not trigger cytokine storm. It helps the body’s natural immune system into action
Other	Amiodarone	It could reduce the internal acidity of endosomes and lysosomes affecting cell activities important for an efficient viral entry
	Bicalutamide, bromhexine, camostat mesilate, nafamostat	Inhibition of TMPRSS2, an enzyme facilitating SARS-CoV-2 cell penetration
	Chlorpromazine	It inhibits clatrine-mediate endocytosis by interacting with dynamin
	Estradiol patch	It could down-regulate ACE2 receptors in kidneys
	Famotidine	It could bind papain-like protease, responsible for initial processing of the SARS-CoV-2 polyprotein into active subunits
	Isotretinoin, retinoic acid	They can down-regulate ACE2 receptors; they are potential protease inhibitors; they could increase CD4 counts
	Ivermectin	It inhibits the replication of SARS-CoV-2 *in vitro*
	Niclosamide	It could block endocytosis of SARS-CoV-2 and prevent its autophagy by inhibition of S-Phase kinase associated protein 2
	Spironolactone	It could, theoretically, reduce ACE-2 expression on lung-cell surfaces
	Verapamil	It could interfere with coronavirus entry and amplification by blocking ion channels

Notes: Drugs involved in safety studies were not included; only drugs approved by regulatory agencies were included. Abbreviations: AAK1, Adaptor-associated protein kinase 1; ACE, Angiotensin Converting Enzyme; COVID-19, coronavirus disease 2019; DPP4, dipeptidyl-dipeptidase four; RdRp, RNA-dependent RNA polymerase; SARS-CoV-2, Severe Acute Respiratory Syndrome Coronavirus 2; TMPRSS2, Transmembrane protease serine 2.

**TABLE 2:** Overview of drugs proposed to potentially counteract the effects of SARS-CoV-2 infection and undergoing experimental studies at July 2, 2020—source: clinicaltrials.gov.

Pharmacological class	Drug	Proposed mechanism in SARS-CoV-2 infection
NSAIDs	Acetylsalicylic acid	It can inhibit virus replication and platelet aggregation, it has anti-inflammatory and could prevent lung injury
	Indomethacin	It could reduce symptoms in COVID-19 patients
	Naproxen	It could reduce symptoms in COVID-19 patients
Kinase inhibitors	Abivertinib, acalabrutinib, ibrutinib, ruxolitinib, anubrutinib	To counteract hyper-inflammatory symptoms caused by cytokine storm
	Pacritinib	It could prevent the development of an inflammatory response to the coronavirus infection and pulmonary failure
Anaesthetics	Isoflurane	In *vivo* studies volatile anaesthetics reduce the severity of ARDS compared to intravenous sedation
	Ketamine	It may be able to interrupt the inflammation that causes COVID-19 symptoms
	Sevoflurane	It has anti-inflammatory properties. In *vivo* studies volatile anaesthetics reduce the severity of ARDS
Antibacterials drugs	Azithromycin, clarithromycin	Macrolides can reduce the inflammatory process and modulate the immune system
Antidepressant drugs	Fluoxetine, fluvoxamine	To counteract hyper-inflammatory symptoms caused by cytokine storm
Anti-thrombotic drugs	Alteplase	Targeting the coagulation and fibrinolytic systems could limit ARDS progression and reduce ARDS-induced death
	Bemiparin, heparin, rivaroxaban, tinzaparin	Prevention of deep vein thrombosis and venous thromboembolism in COVID-19 patients
	Bivalirudin	Potential option to maintain systemic anticoagulation during extracorporeal membrane oxygenation
	Clopidogrel, prasugrel	They could prevent cardiovascular complications in COVID-19 patients
	Defibrotide	To treat endothelial inflammation in severe COVID-19 patients
	Dipyridamole	It could reduce D-dimers concentrations and increase lymphocyte and platelet recovery in the circulation
	Enoxaparin	It reduces D-dimers concentrations and prevents hemostasis abnormalities
	Pentoxifylline	It inhibits the synthesis of pro-inflammatory cytokines; it inhibits platelet aggregation and promotes the fibrinolytic activity
	Plasminogen activator	Targeting the coagulation and fibrinolytic systems could limit ARDS progression and reduce ARDS-induced death
Oncologic drugs	Duvelisib	PI3K inhibition with duvelisib could potentially quell aberrant hyper-activation of the innate immune system, preferentially polarize macrophages, reduce pulmonary inflammation, and limit viral persistence
	Bevacizumab	Suppression of pulmonary edema in COVID-19 patients with ARDS
	Etoposide	To counteract hyper-inflammatory symptoms caused by cytokine storm
	Melphalan	Ultra-low doses of melphalan have local and systemic anti-inflammatory effects and decrease the activation of lymphocytes
	Nivolumab	Nivolumab-induced immunity normalization could stimulate anti-viral response and prevent ARDS development
	Tetrandrine	It could inhibit pulmonary fibrosis
	Thalidomide	It could reduce the persistent cough and reduce the lung damage by blocking the inflammatory response
	Thymalfasin	Administered to individuals with end-stage renal disease, it could reduce the rate and severity of SARS-CoV-2 infection
Antivirals	Isoprinosine	It stimulates a non-specific immune response that is independent of the specific viral antigen
	Maraviroc	It may reverse lymphoid depletion and alter cell trafficking of inflammatory cells, both increasing viral control capacity and dampening damage to lung tissue, respectively
Glucocorticoids	Budesonide, ciclesonide, dexamethasone, hydrocortisone, methylprednisolone, prednisone	To reduce systemic inflammatory response to SARS-CoV-2 infection
Immunosuppressants	Fingolimod	It may confine the over-exuberant inflammatory response and slow down the progress of lung injury
	Levamisole	It can increase lymphocytes and empower the immunity of the body. It can bind to the SARS-CoV-2 protease and can decrease the levels of TNF-α and IL-6
	Methotrexate	It may reduce the over-exuberant inflammatory response and slow down the progress of lung injury
	Olokizumab	To counteract hyper-inflammatory symptoms caused by cytokine storm
	Ozanimod	Its immune-modulating activity could mitigate the morbidity and mortality of COVID-19
Interleukin inhibitors	Anakinra, apilimod, canakinumab, clazakizumab, sarilumab, siltuximab, tocilizumab	To counteract hyper-inflammatory symptoms caused by cytokine storm
Opioids	Naltrexone	It can reduce production of multiple cytokines, inhibit cellular proliferation of T- and B- cells and block Toll-like receptor 4
	Tramadol	It has anti-inflammatory effect decreasing plasma level of TNF-α, which may result in a subsequent increase in T cell numbers
Other	Almitrine	To treat COVID-19-induced hypoxic acute respiratory failure
	Aviptadil	It is a vasoactive intestinal polypeptide (VIP) analogue. In the lug, VIP prevents the activation of caspases, inhibits IL-6 and TNF-α production and protects against HCl-induced pulmonary edema
	Atorvastatin	Inhibition of virus proliferation; it reduced lung virus titers and reduced TNF-α, IL-6 in supernatants of infected cells
	Botulinum neurotoxin	It could attenuate chronic cough, dyspnoea, pneumonia, acute respiratory failure, abnormal circulation, cardiac defects and various neurological deficits
	Cholecalciferol	Its immunomodulatory effects could prevent the occurrence of respiratory derangement and other adverse clinical events
	Colchicine	It reduces cytokine levels as well as the activation of macrophages, neutrophils and the inflammasome
	Conestat alfa	It may dampen uncontrolled complement activation and collateral lung damage and reduce capillary leakage and subsequent pulmonary edema by direct inhibition of kinin-kallikrein system
	Crizanlizumab	It can decrease inflammation by binding to P-selectin, blocking leukocyte and platelet adherence to the vessel wall
	Deferoxamine	Since severe cases of COVID-19 pneumonia have similar clinical presentations of iron overload, it seems that deferoxamine could be a supportive therapy for resolving the complications of COVID-19 pneumonia
	Dornase alfa	It could break down the DNA backbone of neutrophil extracellular traps in the COVID-19 lung which will promote the degradation of pro-inflammatory extracellular histones and prevent the amplification of the inflammatory response and the resultant lung dam
	Eculizumab	It might work as an emergency therapy for the treatment of patients with severe pneumonia or ARDS associated with COVID-19 infection
	Emapalumab	To counteract hyper-inflammatory symptoms caused by cytokine storm
	Human immunoglobulin	It provides passive immune protection against a broad range of pathogens
	Ibudilast	To decrease COVID-19-related acute respiratory distress syndrome
	Iloprost	It may improve inflammation and oxygenation in suspected or confirmed COVID-19 patients with respiratory failure
	Infliximab	To counteract hyper-inflammatory symptoms caused by cytokine storm
	Lanadelumab	Blocking the bradykinin 2 receptor and inhibiting plasma kallikrein activity might have an ameliorating effect on early disease caused by SARS-CoV-2 and might prevent acute respiratory distress syndrome
	Leronlimab	To counteract hyper-inflammatory symptoms caused by cytokine storm
	Lucinactant	It may be able to benefit patients with acute respiratory distress syndrome, improving oxygenation and lung compliance
	Montelukast	It can inhibit the signaling of NF-κB, such as interleukin-6,8,10, TNF-α, MCP-1, and other pro-inflammatory mediators
	N-acetylcysteine	It can reduce the formation of pro-inflammatory cytokines and also has vasodilator properties by increasing cyclic GMP levels and by contributing to the regeneration of endothelial-derived relaxing factor
	Nintedanib	To treat pulmonary fibrosis in patients with moderate to severe COVID -19
	Pamrevlumab	It could reduce edema and block fibrotic degeneration of lung tissue in patients with bilateral COVID-19 pneumonia
	Pirfenidone	It could inhibit apoptosis, down-regulate ACE receptors expression, decrease inflammation, ameliorate oxidative stress and protect pneumocytes and other cells from SARS-CoV-2 invasion and cytokine storm
	Poractant alfa	It could improve oxygenation and survival in COVID-19 patients with acute distress respiratory syndrome
	Prazosin	It prevents cytokine storm and markedly increased survival following inflammatory stimuli in preclinical models
	Progesterone	It could reduce the immunity response
	Pyridostigmine	Acetylcholine-esterase inhibitors may act as immunomodulators during viral infections, potentially reducing the inflammatory cascade observed in critically ill COVID-19 patients
	Ravulizumab	It could counteract hyper-inflammatory symptoms caused by cytokine storm
	Sargramostim	It may confer benefit to patients with ARDS due to COVID-19 exposure, who are at significant risk of mortality
	Sildenafil citrate	It inhibits inducible nitric oxide synthase, an enzyme activating a cascade of inflammatory processes
	Tranexamic acid	It reduces the elevated levels of plasmin/plasminogen
	Ulinastatin	It could counteract hyper-inflammatory symptoms caused by cytokine storm
	Zilucoplan	It can inhibit acute lung injury post COVID-19 and can promote lung repair mechanisms

Notes: Drugs involved in safety studies were not included; only drugs approved by regulatory agencies were included. Abbreviations: ACE, Angiotensin Converting Enzyme; ARDS, Acute respiratory distress syndrome; COVID-19, coronavirus disease 2019; IL, Interleukin; MCP-1, Monocyte chemoattractant protein-1; PI3K, phosphoinositide 3-kinase; SARS-CoV-2, Severe Acute Respiratory Syndrome Coronavirus 2; TNF-*α*, Tumor necrosis factor-alfa.

### Drugs Inhibiting One or More Steps of SARS-CoV-2 Lifecycle

#### Virus Attachment and Entry

The first targetable step of SARS-CoV-2 life cycle is the entry of the virus in the host cells. The virus can enter the cells via endocytosis or via plasma membrane fusion through the interaction between the Spike (S) protein of the virus and angiotensin-converting enzyme 2 (ACE2) and transmembrane protease serine 2 (TMPRSS2) at target cell (Fehr and Perlman, 2015). Specifically, it has been demonstrated that SARS-CoV-2 binds to ACE2 receptors for entry and uses TMPRSS2 for S protein priming, which is essential for the binding to its receptor (Hoffmann et al., 2020). A number of molecules hypothesized to down-regulate ACE2 receptors such as estradiol, spironolactone, isotretinoin and retinoic acid, as well as the TMPRSS2 inhibitors bicalutamide, camostat mesilate and nafamostat were therefore proposed as potential treatments for COVID-19 patients. Furthermore, much attention is being paid to two antiviral drugs potentially inhibiting the binding of SARS-CoV-2 to host cells: umifenovir, which could block the trimerization of the S protein, and nelfinavir, which may bind to the S trimer structure inhibiting the membrane fusion process. It has also been hypothesized that human dipeptidyl peptidase 4 (DPP4) could be a functional receptor for the S protein of SARS-CoV-2, which in turn led to the hypothesis that DPP4 inhibitors may play a role in preventing and reducing the risk and progression of COVID-19 (Iacobellis, 2020). As stated above, SARS-CoV-2 can also enter the cells via endocytosis, a transport mechanism by which the virus is enveloped by the cell membrane and enters the cell within a vesicle. Drugs potentially inhibiting the endocytosis, e.g., the antimalarials chloroquine, hydroxychloroquine, amodiaquine, artemisinin and artesunate baricitinib, chlorpromazine, niclosamide, imatinib and amiodarone and the antimalarials have been therefore proposed to inhibit the entry of SARS-CoV-2 in the host cells. The main proposed mechanisms for the inhibition of endocytosis are: 1) the inhibition of endocytic proteins (e.g., clathrins, adaptor-associated protein kinase 1 and dynamin); 2) the accumulation in acid vesicles and, therefore, the inhibition of viral entry when the endocytosis is pH dependent.

#### Viral Replication

The second step in SARS-CoV-2 lifecycle is the replication of viral RNA from an RNA template, catalyzed by the RNA-dependent RNA polymerase (RdRp) (Fehr and Perlman, 2015). Drugs able to inhibit this enzyme, such as the antiviral drugs favipiravir, galidesivir, tenofovir, sofosbuvir and clevudine, and antivirals inhibiting the replication of RNA, e.g., remdesivir, emtricitabine have been proposed as candidates for repurposing in COVID-19. Interferons (alfa interferon, beta interferon and peginterferon lambda) are also currently being evaluated as viral replication inhibitors and promoters of immune response to clear virus infection. Viral RNA replication is followed by RNA translation and proteolytic processing of viral proteins. Protease inhibitors currently used to treat human immunodeficiency virus and acquired immune deficiency syndrome (HIV/AIDS) such as atazanavir, danoprevir, darunavir, lopinavir and ritonavir and the immunosuppressant levamisole have been therefore proposed as potential treatments to inhibit SARS-CoV-2 replication. Tetracycline derivatives such as doxycycline have also been proposed as candidates for repurposing in COVID-19, due to their potential to chelate metalloproteinases used by coronavirus for cell fusion and virus replication.

#### Virion Assembly and Release

Once the viral structural proteins are synthetized and processed, they are assembled to form new virus particles called virions. They are subsequently transported to the cell surface in vesicles and then released by exocytosis (Fehr and Perlman, 2015). Thus, antiviral drugs acting on this step of viral replication such as oseltamivir and daclatasvir have been proposed for repurposing for COVID-19 treatment, along with other drugs like the immunosuppressant sirolimus.

### Drugs Potentially Counteracting the Effects of SARS-CoV-2 Infection

As stated above, SARS-CoV-2 infection can be associated with amplified immune and inflammatory response leading to an uncontrolled cytokine release, known as cytokine storm, especially in its severe form. The cytokine storm is in turn associated with complications like ARDS, macrophage activation syndrome (MAS), lymphopenia and coagulopathy, representing one of the most studied targets to find an effective treatment for COVID-19 patients (Crisafulli et al., 2020). This is the reason why a number of anti-inflammatory and immunomodulatory drugs like non-steroidal anti-inflammatory drugs (NSAIDs), glucocorticoids, kinase inhibitors and interleukin antagonists are being evaluated to be repositioned in COVID-19. Specifically, these drugs could reduce systemic inflammatory symptoms and counteract cytokine storm effects. Dexamethasone in particular has been evaluated for its ability to counteract the effects of SARS-CoV-2 infection and was the subject of several trials. Indeed, a meta-analysis of seven trials found that dexamethasone was associated with a lower risk of mortality at 28 days (odds ratio = 0.64 [95% confidence interval: 0.50–0.82]) in critically ill COVID-19 patients, compared to placebo or standard of care (Sterne et al., 2020). Anti-inflammatory and immunomodulatory effects have been postulated also for macrolide antibiotics such as azithromycin and clarithromycin (Sultana et al., 2020a).

To treat respiratory complications due to SARS-CoV-2 infection such ARDS, several drugs approved for the treatment of idiopathic pulmonary fibrosis such as nintedanib, pirfenidone and pamrevlumab have been proposed. Other drugs currently being evaluated as candidates for repositioning in COVID-19-induced ARDS are bevacizumab, aviptadil, eculizumab and conestat alfa, due to their potential to reduce pulmonary edema. Coagulopathy is another of the main complications of COVID-19-triggered cytokine storm, suggesting the potential role of anti-thrombotic agents to prevent venous thromboembolism. Coagulation is activated by the inflammatory response through several pro-coagulant pathways (Connors and Levy, 2020) and it presents with a considerable increase of D-dimer levels that could be ascribable to the attempt of the fibrinolytic system to remove fibrin and necrotic tissue from the lung parenchyma (Medcalf et al., 2020). Indeed, it has also been suggested that fibrinolytic therapy may be an effective pharmacological strategy to treat acute lung injury in COVID-19 patients (Liu et al., 2018). Based on these considerations, tissue-plasminogen activator and alteplase are currently being investigated in experimental studies for repurposing in COVID-19 patients. Finally, it is interesting to note that some approved drugs with mechanism of actions that are not immediately associated with COVID-19 have also been proposed as candidates for repositioning. Examples include general anaesthetics ketamine, sevoflurane and isoflurane, which are hypothesized to reduce systemic inflammation and ARDS severity. Further examples include the antidepressants fluoxetine and fluvoxamine, which are thought to be potentially able to counteract hyper-inflammatory symptoms caused by cytokine storm. Another drug proposed as a potential therapy for COVID-19, although not yet evaluated in a clinical trial, is the mood stabilizer lithium, whose antiviral activity was demonstrated at preclinical level, but it was not confirmed in clinical settings (Murru et al., 2020; Rajkumar, 2020).

## Sources of Evidence for the Benefit-Risk Evaluation of Drugs for Repurposing in COVID-19

### Pre-Clinical Studies

Pre-clinical studies are those studies which are conducted *in vitro* (in a pathogen, animal or human cells) or *in vivo* through animal models prior to the initiation of clinical studies in patients, whether healthy or sick. They include studies which are conducted in silico (Elmezayen et al., 2020), *in vitro*, such as receptor-binding assays to identify drug ligands and studies using cell lines or tissues excised from animals or humans representative of a specific disease, to evaluate how these biological milieus of varying complexity respond to a drug. Pre-clinical studies also include those studies which are conducted *in vivo*, where animals are exposed to a particular drug. The animals treated with a drug are then tested for various biological and/or behavioral parameters and may be sacrificed for histological examinations. Such animals may be small models, such as mice or ferrets, or larger models, such as non-human primates. A compendium of existing animal models used for COVID-19 research is available in the public domain (National Center for Advancing Translational Sciences, 2020). Taken together, pre-clinical studies can be useful in drug repurposing in the phase of hypothesis generation. Through these studies, the pharmacological mechanism of a drug in a novel context, in this case in SARS-CoV-2 infection can be better understood. This is important to attribute a degree of biological plausibility to the hypothesized effect of a drug in a new indication, as indeed, all indications of repurposed drugs are *de facto* new.

Ideally, pre-clinical studies in a repurposing context should specifically concern the effects of a drug in the disease for which repurposing is being attempted. For example, pre-clinical studies to support the repurposing of an anti-viral drug for SARS-CoV-2 should be conducted using microbiological assays, or cell lines and animal models which have been infected with the agent of interest, i.e., SARS-CoV-2. However, this presupposes an accurate knowledge of the disease pathology and symptoms, which may be problematic in a novel disease such as COVID-19. If the biological and clinical aspects of the disease which are critical determinants of clinical outcomes are not known, they cannot be measured and identified as therapeutic targets, even in a pre-clinical setting. Direct pre-clinical evidence of the infectious agent of interest may also be limited during a pandemic for several reasons. First, the virus has to be isolated, described and cultured before it can be sent to laboratories, unlike other microbial agents which are already known and available *in vitro*. Secondly, while *in vivo* experiments are higher up the evidence hierarchy than *in vitro* experiments because they represent a complex living organism rather than isolated cells of tissues, the pathology of SARS-CoV-2 may be different in animals as compared to humans. As a result, the safety and efficacy of drugs *in vivo* may not be generalizable to humans. Some animal models may need to be specifically developed to address a particular hypothesis and may not be available on large scale. One example is the transgenic mouse model which produces the human ACE2 protein, needed to conduct pre-clinical studies concerning renin-angiotensin-aldosterone system (RAAS) inhibitors, such as angiotensin converting enzyme inhibitors (ACEIs) and angiotensin receptor blockers (ARBs) (Callaway, 2020). Once developed, the effect of the virus in the mouse model needs to be described in detail for it to be accepted as a valid model of the disease (Bao et al., 2020; Jiang et al., 2020). Only then can pharmacological testing be considered potentially informative. In the absence of *in vitro* and *in vivo* models of SARS-CoV-2, models of other related infective agents can be and have been considered but their relevance to the disease of interest may be ambiguous. An example is how to support the biological plausibility of azithromycin, an antibiotic, being clinically useful in COVID-19, caused by a virus, is based on its ability to modify the reaction of the immune system and to reduce inflammation (Kanoh and Rubin, 2010; Parnham et al., 2014; Cramer et al., 2017) in a pre-clinical setting. This information was not derived directly from SARS-CoV-2 infections. The effectiveness of azithromycin in a clinical setting has not been proved to date (Sultana et al., 2020a).

Pre-clinical studies can be useful because they can provide results in a relatively short time compared to clinical studies concerning whether a particular pharmacological approach is likely to be worth pursuing. However, the information they can provide on drug safety and efficacy is very limited because pre-clinical studies necessarily provide an incomplete picture of disease pathology as well as potentially generating contrasting findings. The real value of pre-clinical evidence must be proven in a clinical setting. ACEIs and ARBs are a good example of how pharmacological evidence derived from a pre-clinical setting is not always directly translatable to the clinical setting. Pre-clinical evidence on these drugs was not consistent to begin with, with some research suggesting that based on their pharmacological interaction with the virus, that they will worsen prognosis after SARS-CoV-2 infection and/or increase the risk of infection (Fang et al., 2020; Gurwitz, 2020; Zheng et al., 2020), and other research findings concluding that they are likely to be protective (Gurwitz, 2020). Ultimately, in several observational studies of infected patients it was seen that ACEIs and ARBs are likely to be neither (Li et al., 2020a; de Abajo et al., 2020; Feng et al., 2020; Fosbøl et al., 2020; Gao et al., 2020; Mancia et al., 2020; Mehta et al., 2020; Meng et al., 2020; Reynolds et al., 2020; Selçuk et al., 2020; Yang et al., 2020; Zhang et al., 2020). Another example concerns hydroxychloroquine, which was found to inhibit SARS-CoV-2 *in vitro* (Liu et al., 2020) but not found yet to be effective in clinical practice (Geleris et al., 2020), either as treatment or as post-exposure prophylaxis (Boulware et al., 2020). Another important role of pre-clinical studies is to evaluate the pharmacokinetic profile of a potential drug candidate, in order to ensure that the effective doses in animal models can be translated safely to humans.

### Clinical Trials

Clinical trials, ranging from single-arm open-label trials to randomised controlled trials (RCTs) provide information on drug tolerability and, most importantly, efficacy. RCTs are considered the gold standard of evidence generation on drug efficacy because randomization of treatment randomly distributes potential confounders among treated and untreated patients. The masking of participants, investigators, and/or outcomes assessor to treatment assignment further increases the reliability of results by preventing bias. The clinical information that trials can provide concerning potentially repurposable drugs for SARS-CoV-2 varies widely and can refer to very specific indications such as post-exposure prophylaxis (NCT04321174 - lopinavir/ritonavir RCT), treatment of mild COVID-19 (NCT04342663 - fluvoxamine RCT) and treatment of hospitalized patients with COVID-19 (NCT04280705 - remdesivir RCT) as well as critically ill hospitalized patients specifically (NCT04244591 - methylprednisolone RCT). This has implications for the generalizability of study results and the application of findings in clinical practice. To address this issue, both the FDA and the World Health Organization (WHO) have published guidance on the recommended standards in drug development and clinical research in COVID-19 (Food and Drug Administration, 2020b; Marshall et al., 2020b).

Although serendipitous drug rediscovery is possible, it is perhaps more likely that drugs with a consolidated pharmacological target in the disease of interest have a higher chance of successfully getting through trials, not least because this may impact the choice of appropriate outcomes. An example of a drug which is not supported by a strong pharmacological rationale is fluvoxamine, currently being tested for the treatment of non-hospitalized persons with COVID-19 (NCT04342663 - fluvoxamine RCT). Another example is estradiol, where evidence is still emerging (Li et al., 2020b), currently being tested among persons with suspected or confirmed COVID-19 to evaluate whether it reduces the severity of symptoms (NCT04359329 - estradiol patch RCT). These examples highlight the complementarity of pre-clinical and clinical studies in drug repurposing as in drug discovery, because pre-clinical evidence can suggest the biological plausibility of a drug being effective while the clinical evidence is needed to confirm that a drug is effective and safe.

Conducting methodologically sound trials during a pandemic can be challenging, for scientific, logistical and ethical reasons (Angus, 2020) and rapid dissemination of poor quality studies can have serious consequences (Kim et al., 2020). Some issues highlight the need to interpret the available clinical trials with great care. Not all trials are created equal and there is an accepted hierarchy of quality. Open-label trials, such as an ongoing trial for ruxolitinib in combination with simvastatin (NCT04348695 - ruxolitinib in combination with simvastatin RCT) in such a highly charged situation as a pandemic may lead to bias, as compared to double-blind trials (Wang et al., 2020). Similarly, clinical studies lacking randomization (NCT04304313 - sildenafil non-randomized trial) are considered much less reliable than studies randomizing treatment (NCT04350593 - dapagliflozin RCT).

Patient recruitment can be more problematic when organizing a trial during a pandemic than it would otherwise be. The size of a study population can limit the scope of causal inference if a trial is underpowered. There are currently ongoing trials to repurpose drugs which aim to recruit as little as 10 patients (NCT04304313 - sildenafil non-randomized trial). Restrictive inclusion and exclusion criteria may not only lead to an under-powered study but also to a patient population that is not representative of the real-world setting. For example, in a randomized double-blind trial of fluvoxamine in COVID-19, patients with severe underlying respiratory disease were excluded, as were patients with dementia, as these could not provide informed consent (NCT04342663 - fluvoxamine RCT). A broader population would only be included in phase III trials. While such exclusion criteria are necessary for the purpose of causal inference and to respect patients’ right to self-determination, lack of this data in phase III confirmatory trials may limit the generalizability of study findings by excluding patients who are at highest risk of contracting COVID-19 and/or developing severe symptoms as well as of developing adverse drug effects. Even patient follow-up can be problematic during a pandemic. Some researchers have resolved this issue by conducting trials remotely, for example completely avoiding face-to-face encounters, with study material, including the study drug, being delivered to patients’ homes (NCT04342663 - fluvoxamine RCT). This could be a problem for taking into account patient compliance and is likely to be more viable for cases of COVID-19 which are treated at home, however use of digital communications such as text messaging, teleconference programs and couriers has largely overcome this. While ideally a clinical trial should be conducted according to a strict protocol, there are ethical implications in continuing to treat patients with a comparator drug if the main study drug appears to be effective. Indeed, some trials explicitly state that should interim results suggest a study drug is effective, it will be considered the new control (NCT04280705 - remdesivir RCT). Small controlled trials are considered to be less useful when evaluating drug safety but this is potentially less of a problem with drugs which have already been approved, because their safety profile is likely to have already been well-described. Indeed, this is the main advantage of repurposing drugs as opposed to *de novo* drug discovery. However, because of their limitations clinical trials must be complemented by observational studies conducted in a real-world setting.

A variant of clinical trials worth mentioning, a hybrid between trial design and observational study design, is the adaptive trial. In adaptive trials, a review and adapt approach is used while the trial is being conducted, as opposed to the linear approach used in classical trials, where the trial occurs in three distinct phases, i.e., trial design, implementation and analyses (Pallmann et al., 2018). As a result, certain changes can be made to trial design and analytic plans based on preliminary data. Such a study design can be very valuable in the pandemic setting because new findings are constantly emerging and standards of care are rapidly changing. Some examples of adaptive trials being carried out to evaluate drug efficacy and safety for COVID-19 treatment include the REMAP-CAP (Angus et al., 2020), WHO SOLIDARITY trial (World Health Organization, 2020) and RECOVERY trials (Horby et al., 2020).

There is a notable interplay between regulatory and government bodies and researchers conducting clinical trials, as such entities have a role in regulating clinical trials. An example is the ACTIV/Operation Warp Speed groups in the United States that are coordinating pre-clinical studies and clinical evaluation of interventions to advance the most promising candidates given the limited resources available, including animals for pre-clinical evaluation and patient population for clinical evaluation.

### Observational Studies

While clinical studies are useful to evaluate drug efficacy, they may not be able to provide evidence rapidly and on a large scale, as they are contingent on the speed of prospective data collection and analysis. During a pandemic, time is of essence and public health authorities cannot wait for weeks until trial results get published. Observational studies have an important role to play in this regard because they rely either on data which has already been collected or on data which is collected quickly in a prospective manner using previously established data systems and can therefore be conducted rapidly. They should be conducted alongside prospective controlled clinical trials which establish pharmacological basis of evidence. The main role of observational studies in evidence generation concerns drugs which are used off-label for COVID-19. For example, a retrospective observational study was carried out in a hospital in Lombardy, Italy, to evaluate the effectiveness of anakinra in improving clinical outcomes among patients infected with COVID-19, finding that this IL-1 antagonist was effective (Cavalli et al., 2020). Similarly, an observational study was conducted in hospitals of the Bologna and Emilia-Romagna areas in Italy, to assess the effectiveness of tocilizumab in improving clinical outcomes among patients with COVID-19, also finding that this drug is potentially effective (Campochiaro et al., 2020). It was also observational studies which were able to rapidly shed light on lack of effectiveness of RAAS inhibitors in improving clinical outcomes in COVID-19 (Li et al., 2020a; de Abajo et al., 2020; Feng et al., 2020; Fosbøl et al., 2020; Gao et al., 2020; Mancia et al., 2020; Mehta et al., 2020; Meng et al., 2020; Reynolds et al., 2020; Selçuk et al., 2020; Yang et al., 2020; Zhang et al., 2020). Another example yet is an observational study which showed that drugs used to treat prostate cancer, androgen-deprivation therapies, may have a protective effect among persons with COVID-19 (Montopoli et al., 2020). Observational studies have other advantages in addition to the speed of data collection and large sample sizes. The populations identified in these studies are a reflection of real-world patients, including those patients who are likely to be excluded by trials—children, the very old, persons with serious underlying respiratory diseases and persons with a large number of concomitant medications (Sultana et al., 2013).

To date, there are 50 studies concerning COVID-19 recorded in the EU post-authorization study (PAS) register, the official European repository of PAS. The PASs for a repurposed product in a pandemic depend on the potential and identified risks, as well as the important missing information identified during pre-marketing. While the gaps in the safety and effectiveness information at the time of licensing are greater for a repurposed product that has been developed expeditiously, the principles are similar to conventional products. Research questions need to be formulated based on the public health needs and the available pre-marketing data. The next task will be selecting the most appropriate study approach (primary or secondary data collection), study setting or data resource and study design (cohort, case-control, nested case-control, cross sectional or in some cases simple randomised clinical trial). Open extensions of post-marketing clinical trials are an important method which will be used frequently. Situations where the incidence and prevalence of cases is low present a challenge; a solution is to conduct the studies in countries with higher incidence of the infection or extending the study population by conducting multi-country studies. The sample size of studies spanning several countries is typically larger than those restricted to a single country, meaning that the target recruitment is generally achieved more quickly and results are available faster. Multinational studies do not only report the effect of the drug but also reflect the nature and delivery of the healthcare system in the different countries. This in turn has an impact on the conduct of the studies, as certain types of health information may not be available in the same way in all study countries. This includes information such as exposure, outcomes, confounders and previous medical history. However, this is not an insurmountable issue because one of the key points of studies conducted in a network of countries is that the master study protocol can be modified to take account of different data sources. Depending on the heterogeneity or similarity of the information available, results can be meta-analyzed or pooled with data from different sources with the same objectives. Multi-database studies are becoming increasingly important as their potential in reaching large sample sizes over a short observation period is recognized (Gini et al., 2020). Indeed, it is internationally acknowledged that having a stable multi-database network to rapidly provide almost real-time healthcare information to regulatory and public health agencies is very important (Sultana et al., 2020b). This potential is highlighted in a public health crisis such as the COVID-19 pandemic.

Of course, observational studies also have their limitations. Since they are often conducted using sources of secondary data, i.e., data not intended primarily for research purposes, the quality of the data must be thoroughly checked as it can contain errors in data entry, such as incomplete data, Anatomic Therapeutic Chemical (ATC) classification codes at a lower level than the fifth level, missing data, such as sex, or clearly implausible data, such as date of birth set in the future. Furthermore, diagnosis data in secondary data sources may be of limited reliability and there may be underestimation of certain diseases based on the context in which coding is assigned. For example, acute conditions are likely to be better captured in secondary data sources such as hospital claims while chronic conditions are likely to be better captured in primary care electronic records (Trifirò et al., 2019). In the case of COVID-19 specifically, observational studies may be limited by the extent to which they can reliably identify patients who have a microbiologically ascertained diagnosis of COVID-19. Finally, unlike classical clinical trials, observational studies cannot ascertain that a prescribed or dispensed drug was truly administered. This is assumed to be the case, but in the context of a pandemic, it is possible that patients may not actually go to fill their prescriptions.

## Conclusion

Several drugs are currently being studied as potential repurposing candidates in clinical trials, but to date only remdesivir has been approved for the treatment of COVID-19. Although drug repurposing has the potential to decrease the time usually required for a drug to reach the market, it is a process that is still associated with many challenges, whether from a regulatory or a scientific perspective. Close collaboration between various stakeholders is needed to leverage and critically evaluate existing evidence and strategically plan the generation of new pre-clinical, clinical and observational evidence to investigate the efficacy/effectiveness and safety of drug for potential repurposing. One of the main objectives of such a collaboration should be to avoid duplication of studies and plan studies in such a way that the outcomes evaluated can be compared. Pre-clinical, clinical and observational research all generate complementary information which is necessary in building the case for drug repurposing.

## Author Contributions

GT conceived the study. JS, SC, FG, EL, and SS wrote the first draft of the paper.

## Conflict of Interest

Author FG was employed by the company tranScrip.

The remaining authors declare that the research was conducted in the absence of any commercial or financial relationships that could be construed as a potential conflict of interest.
